# From traditional to transactional: exploration of khat use in Ethiopia through an interpretative phenomenological analysis

**DOI:** 10.1186/s12889-024-19357-1

**Published:** 2024-07-15

**Authors:** Elizabeth A. Wood, Stuart J. Case, Sarah L. Collins, Heather Stark, Tara Wilfong

**Affiliations:** 1 Eck Institute for Global Health, University of Notre Dame, 921 Flanner Hall, Notre Dame, IN 46556 USA; 2 Department of Epidemiology, College of Public Health & Health Professions, University of Florida, 1225 Center Drive, Gainesville, FL 32611 USA; 3 Department of Health, Sport & Exercise Sciences, School of Education & Human Sciences, University of Kansas, Lawrence, KS USA; 4 School of Public Health, College of Health and Medical Sciences, Haramaya University, Harar, Ethiopia

**Keywords:** Khat, Ethiopia, Interpretative phenomenology approach, Qualitative research

## Abstract

Khat, a naturally growing stimulant, has seen a significant increase in both consumption and cultivation in eastern Ethiopia. This reliance on khat in the region comes despite its known physiological complications, with users unable to restrict khat use due to its pervasive impact on their livelihood. This qualitative study sought to understand the meaning that those in eastern Ethiopia attribute to khat and explore their firsthand experiences with the substance. In June and July of 2023, six unstructured interviews were conducted among residents of the Haramaya District in Ethiopia. To promote a holistic comprehension of the participants’ lived experiences, an interpretative phenomenological analysis approach was employed when collecting and analyzing the data. Participant responses were coded independently from one another by two different researchers identifying superordinate and corresponding subordinate themes. Among the participants, six superordinate themes were captured: economic backbone of the region, market disruption & fluctuation, pesticide use, societal relationships around khat, applications of khat, and access to healthcare. The participants’ responses indicated that the normalization of khat use, coupled with the downplaying of its addictive potential, has established a framework where khat consumption is not only allowed but, in some cases, even encouraged. The unique interplay between communal practice and individual preservation creates a cyclical effect of using khat to supplement energy to farm khat and then sell or stimulate further work on their farm. This study illuminates the transitionfrom what was once the traditional or spiritual use of khat, to a more practical use for ensuring economic livelihood.

## Background

Khat is a natural stimulant grown in eastern Africa and the Middle East that comes from the *Catha edulis* plant [1]. Khat’s chemical composition is similar to amphetamines, containing the compound cathinone which produces euphorigenic effects and stimulation [1, 2]. As such, khat can be addictive, with those chewing it recognizing their dependence on the stimulant to complete everyday tasks [3, 4]. Risk factors that increase one’s likelihood of chewing khat include identifying as male, having family members and peers who also chew khat, smoking frequently, having previous traumatic experiences, and living in either Oromia or Harar Regions in Ethiopia [3, 5, 6]. Those who use khat explain their use is primarily driven by its ability to increase concentration and to socialize with their friends and family [7].

Despite the proliferation of khat use in East Africa, the stimulant has been associated with numerous mental and physical complications for those who use the substance. In considering the cognitive effects of khat chewing, active khat chewers were significantly more likely to be either depressed or have anxiety when compared to nonchewers [8]. Likewise, increased consumption of khat is associated with an increase in the number of post-traumatic stress disorder (PTSD) symptoms experienced [3]. These psychological consequences of khat are not solely associated with just the consumption of khat but also the intensity of khat use, as those who use khat more frequently are more likely to report mental distress [9]. Physically, khat has been shown to cause a variety of gastrointestinal disorders, including constipation, gastritis, and hemorrhoids [10]. Those who chew khat also experienced an increased prevalence of hypertension as well as increasing one’s risk for acute myocardial infarction [11]. Given the severity of both the cognitive and physical symptoms inherent to khat use, there is a concern that the proliferation of the substance will further worsen the health of those who use khat, resulting in both negative personal and broader population health consequences.

In one study in southern Ethiopia [7], among those who chewed khat, 68.4% chewed daily. This addiction to khat is advanced by the short duration of the effects of khat use, with the positive effects of improved well-being and stimulation lasting only a few hours [12]. As a result of the short-lasting benefits, those who use khat are driven to use the substance more regularly to sustain its physical and psychological impacts. Another factor contributing to the addictive nature of khat is the severity of withdrawal symptoms, with those who discontinue use for an extended period experiencing mood disturbances, headaches, and general irritability [8]. As such, those who chew khat would rather continue to use the substance rather than endure the arduous withdrawal process. Even those who are aware of and have experienced these withdrawal symptoms still chew khat, as they perceive the benefits of use as outweighing its consequences [13].

Although many people who chew khat recognize their dependency on khat for daily activities [13], the stigma surrounding addiction prevents people who chew khat from addressing their addiction. Those who use khat in Somalia noted how they do not believe in an addiction to khat, which can limit their proclivity to address their reliance on it [14]. Given that Islam is predominantly practiced in both Somalia and eastern Ethiopia, it is critical to acknowledge the prohibition of substances deemed intoxicants according to the religion [15]. Bearing this perspective in mind, it is intelligible that those who use khat do not view their khat use as addiction due to the negative connotation coinciding with addiction.

One possible explanation for the prevalence and intensity of khat use in Ethiopia is how dependent both citizens and the country as a whole are on the sale of khat [16]. The khat trade is a considerable way for local farmers in eastern Ethiopia to make money and serves as a major agricultural crop in the region [17]. Depending on the region of Ethiopia, households can spend a significant amount on khat, which also translates to a significant amount of earnings from khat sales contributing to a household’s income as well as the Ethiopian economy. Khat was the fourth most exported good in Ethiopia, accounting for 11.1% of domestic exports in 2021, amounting to 402.5 million USD [18]. In a survey of Ethiopian farmers, it was reported that more than half of their total income could be attributed to khat [19]. In a recent study in Haramaya District [20], almost all households surveyed grew khat (95.3%), with one-fourth of the participants not spending any money on khat due to their own cultivation. In contrast, in other regions of Ethiopia where khat is not as widely grown at the household level, a significant portion of household income is spent on purchasing the substance. A survey of 359 Ethiopians who lived west of the capital Addis Ababa found that on average they spent 790 birr per month [8]. This accounts for what could be a low-paying government salary within Ethiopia.

Khat consumption and cultivation in Haramaya, Ethiopia, is widespread and culturally embedded, largely attributed to its socio-economic and cultural significance in the region. It is deeply integrated into the social fabric of Haramaya, often used in social gatherings, religious ceremonies, and as a means of facilitating conversation and community bonding [20]. The farming of khat is a significant economic driver in the area, providing livelihoods for many farmers and traders due to its lucrative market, both locally and internationally. Despite the immersion of khat within Ethiopia, there is limited research as to why khat has proliferated in such a rapid manner and how khat has impacted both society and the daily lives of Ethiopians. As such, this research aimed to explore the firsthand experiences of individuals with khat within the Haramaya District (within the Oromia Region of eastern Ethiopia), given khat’s significance on one’s livelihood for those living in the area. The study aimed to gain an understanding of the livelihoods and the physiological, social, and economic consequences associated with khat consumption and khat cultivation. A particular emphasis was placed on delving into the meaning participants attribute to khat within this region, its functions within society, its influence on social norms, and the overall impact on their daily lives.

Reflexivity Statement.

The primary author and one of the two researchers who analyzed the data have a background in qualitative design, methods, and analysis. She also has a background in religion, specifically Islam, as well as a personal relationship with substance abuse disorders. Other authors that assisted with analysis have backgrounds in qualitative research, live specifically within the catchment area where the research took place, and/or have a personal experience with khat use within Harar, Ethiopia.

## Methods

### Study design

This qualitative case study employed unstructured in-depth interviews with an interpretative phenomenological analysis (IPA). The choice of IPA was deliberate, as it aligned with the study’s objective to comprehensively understand the meaning associated with the physiological, social, and economic experiences of individuals engaging with khat from various perspectives. The data collection method of choice was unstructured interviews because they facilitated the development of a strong connection and an in-depth exploration of the individuals’ experiences, thoughts, emotions, and sense-making [21]. The participants guided the conversation’s trajectory, while the interviewer also formulated questions based on their narrative. This structure allows for parity between maintaining some consistency among interviews while allowing flexibility and fluidity within each conversation [22]. Based on the phenomenological approach’s focus, the study did not attempt to achieve generalizability in the results but instead sought to capture a level of shared experiences among participants [23].

The study was approved by the University of Florida Institutional Review Board (IRB# 202,300,400), the University of Notre Dame Institutional Review Board (22-11-7493), and Haramaya University’s Institutional Health Research Ethics Review Committee (EFP 2023–3–08). Written informed consent was obtained from the study participants after explaining the study’s purpose, procedure, duration, risks, and benefits.

### Participants & data collection

Purposive sampling was adopted to recruit participants from the Haramaya District of eastern Ethiopia between June and July of 2023. Eligibility criteria included being at least 18 years of age, living in the Haramaya District for at least six months, and having experience with khat in some capacity including but not limited to khat use, khat cultivation, and witnessing the health effects of khat.

Face-to-face unstructured interviews were conducted among the six participants who provided written consent to participate in this study. Four of the six interviews were carried out in English, while the other two required a translator who spoke both Afan Oromo and English fluently and would translate in real time. The interviewer had a background in qualitative research methods and analysis, as well as conducting face-to-face interviews on potentially sensitive topic areas. Interviews were audio recorded with permission from the participants and lasted on average 30 min. Interviews took place primarily indoors, however, khat farmers were interviewed on their property at their request to better describe their khat farm. All audio recordings were uploaded to Otter.ai for transcription purposes with the interviewer present for all interviews checking the transcriptions for quality control. All of the interviewer’s notes, memos, and observations were also used for analysis in accordance with interpretative phenomenological transcription.

### Data analysis

Interviews were analyzed using an interpretative phenomenological analysis guided by Biggerstaff and Thompson [24] as well as Lincoln and Guba [25] to ensure different elements of trustworthiness throughout the analysis process. Following each interview, the recordings were transcribed fastidiously, incorporating details such as pauses, disturbances, grammatical mistakes, and speech dynamics [24, 26]. The data underwent a thorough, independent review by two of the study researchers. The two researchers engaged in bracketing as a method to enhance the trustworthiness of their study by systematically reflecting on and setting aside their own biases. Bracketing, or epoche, has been integral to phenomenology since its inception [27]. Additionally, the researchers held regular peer briefings to discuss their reflections and challenge each other’s assumptions. This collaborative dialogue helped to identify and mitigate any biases that could influence the interpretation of the data. By openly acknowledging and discussing their subjective perspectives, the researchers aimed to maintain a critical awareness of how their backgrounds and beliefs might shape the research outcomes.

Emerging themes were organized into superordinate themes along with corresponding subordinate themes. This method was applied to each participant individually, treating each case with attention to its unique perspective around khat and setting aside themes and interpretations from one case while analyzing another. Acknowledging each researcher’s subjective interpretative role and aiming to anchor interpretations in the transcripts, themes underwent negotiations among the two researchers analyzing the data to ensure agreement of final superordinate and subordinate themes.

## Results

Six participants were included in this study. Pseudonyms were used to represent the sex and ethnicity of the participants (Table 1). Six superordinate themes were captured from the interviews with ten corresponding subordinate themes (Table 2). Results are presented in no particular order.

**Table 1:** In-depth interview participant descriptions

Participant Pseudonym	Sex	Occupation
Adnan	Male	Development
Mariam	Female	Government Worker
Abdul	Male	Khat Farmer
Ahmed	Male	Khat Farmer
Abbas	Male	Khat User/Entrepreneur
Hasan	Male	Microbiologist

**Table 2:** Superordinate and subordinate themes

Superordinates	Subordinates
Economic backbone of the region	
	Khat is the most cost-effective cash crop
	Purchasing and selling khat; differences between men and women
Market disruption & fluctuation	
	Reliance on exportation
	Unpredictability of the market
Pesticide use	
	Stigma of using pesticides
	Runoff exposing people through drinking water
Societal relationships around khat	
	Transition of khat use
	Societal pressures
Applications of khat	
	Physiological experiences
	Differences in rural versus urban experiences
Access to healthcare	

### Economic backbone of the region

Several participants discussed khat being an essential component of the Haramaya and Harar economies. So much so that Adnan describes how, “Khat is a big, the second cash crop in this area and the main livelihood for the rural community in this area. So, the backbone for the rural community or the economic backbone of the community.” Due to its emphasis as the “backbone,” unlike other areas, where coffee and other crops may be dominant, Harar has developed a reputation for harvesting the best quality khat in the region.

### Khat is the most cost-effective cash crop

*Abbas*, an active user of khat and self-proclaimed entrepreneur, recognized that khat currently offers the best return on investment. This was described as,

And the people, the farmers following the, you know, the income, what kind of crop or product will generate income for us they’re following that one. That’s why, for example, if the income of potato, or income of sorghum or maize will be greater than the khat income, they will leave this one [points to khat] and will turn their cultivating to the highest income-generating product. Since the demand…the demand of the khat and the price, income is very high, having certain, you know, cultivating or plantings around this area.

Here, Abbas expressively depicts that the increased demand for khat ultimately perpetuates its continued production and use compared to other crops, which is particularly beneficial in eastern Ethiopia where most people have small plots of land that must be used judiciously. Comparatively, Abdul, a local khat farmer, shared his perspective stating,

Well, when you compare to other crops, khat [has] high prices. Yeah. And then the farmer may sell it for four times per year. Yeah, yeah. So, for example, we take the coffee, coffee is harvested once per year. This [khat] is like three or four times per year.

Abbas also describes how if a different crop offered the same return on investment, then farmers in Harar and Haramaya would shift to that crop. Collectively, Abbas’s and Abdul’s accounts demonstrate that khat yields a high-return crop option that bolsters income and economic work.

### Purchasing and selling khat; differences between men and women

In eastern Ethiopia, specifically Aweday City, the khat town between Haramaya and Harar, men often load khat into large trucks that will export the khat to countries such as Djibouti and Somalia. Unlike other areas of Ethiopia, women are also seen in this city selling khat at the local level. There were also differences observed regarding those who grow and cultivate the khat. For example, Adnan shares, “Yeah, khat planted by the mens.” He then continues by stating,

And then it grows to reach two to three years at the start of harvesting. So, there is different types of khat harvesting: market-based; household-level consumption; domestic market consumption. The [first] one is the export market which is high value that is owned by men and sold by men and take[n] care of by men. While the other is by woman and children. So, the yield for the basic income for the household consumption and utilities and the like were [from] the harvesting of the market only maybe two to three per year.

Here, Adnan is able to articulate the distinct differences in harvesting and consumption practices rooted in gender norms. Interestingly though, Adnan also described how men and women spend the money from khat sales differently. It was noted that women use money from khat to contribute to the household food and other needs; however, Adnan would not answer how men spend their money from khat. Abbas provides insight into how women selling khat is unique to this region:

Such a trend is you know now in Ethiopia and some you know selling khat is really easy work not hard work. That’s why womans are selling, but men also can do it, but 99.9% is woman. But if you are going to visit Djibouti khat market, the majority of them are men. Men will sell the khat, in Somalia also. Even in Addis Ababa, if you go, all who sell the khat is a man, not a woman. Around this area peoples are separating traditionally from that.

Abbas clarifies that while women selling khat in this region is not uncommon, it is something that was adopted demonstrating the evolving gender and social norms and divergence from traditional practices. This shift may be due to the abundance of khat in the region since Abbas also states women in other areas of Ethiopia do not engage in selling khat.

### Market disruption & fluctuation

Several kinds of market shocks and disruptions were discussed, including seasonality, general supply and demand, as well as the ability to export khat to other surrounding countries. Khat also requires a lot of water to grow which can become a challenge during prolonged droughts. Adnan describes the capricious nature of khat:

Yeah, the supply is actually different from season to season when the rainy season all areas of the farmers get the khat. So, the volume, the supply to the market increases [which is] the reason the price decreases, but it is still because of different variet[ies] in the quality the prices vary. For generally the supplies increase, then the price is reduced.

Adnan summarizes how the cost of khat fluctuates for various reasons, including both seasonality and quality of khat. This, in turn, creates unpredictable markets for khat within the region that can impact livelihood.

### Reliance on exportation

Several participants characterize khat as an essential part of the eastern Ethiopian economy largely because they can sell it to the surrounding countries of Djibouti and Somalia - where demand and the price of khat are high. For example, Abbas notes,

And this khat is, you know, economically important for the eastern part of Ethiopia. It’s estimated, you know, you know, 90% of Ethiopia, eastern Ethiopia is covered by khat. The purpose of you know, cultivation of khat, or planting the khat in this part is geographically close to the eastern part [of] Somalia and Djibouti. Somalia and Djibouti is most you know, a big market for our khat markets. And as the demand is very high.

This description not only indicates an economic reliance on exportation but also depicts an element of connectivity with neighboring communities and an expected reliance on khat distribution across these communities. Diminishing the availability or practice of khat in this region is therefore not only tied to economic stability but also partnership and relationality.

### Unpredictability of the market

Throughout this study’s data collection, it was conveyed by several people that the price of khat locally had decreased precipitously. It was unclear why the supply was so high in the local market, plummeting the price of khat. When probed, some participants refused to answer the question and others stated they did not know. For example, one participant was distracted and when asked the question again waved it off to move on. Another participant, when asked if there had been any changes in policy or government regulations, shrugged and specifically noted that they ‘did not know about the government.’ However, Abbas offered his perspective that it was driven by the government:

This one [points to khat in the center of the room] is now 2000 birr. This one. When it [the market] is good, expensive. The market for khat in this year is very low. But when it’s a good time for khat, this amount is 10,000…0.10,000 [birrs]. For example last year, last year this amount was 10,000. Now it is 2000, now buy it for 2000…Today, this year there is a government policy. We’ll grow khat and be taxed, but, and it’s not supported by the government. There is not support for the khat and the markets.

This insinuation of government involvement may be a direct factor of why others were hesitant or resistant to sharing more.

### Pesticide use

Pesticide use, or chemicals as some Ethiopians called them, was a contentious topic for several of the participants, specifically the khat farmers because they did not want their farms linked with using pesticides to supplement khat cultivation. Regionally, chemically treated khat is viewed as lesser quality than organic khat, thus being perceived as or using pesticides could potentially be detrimental to a farmer’s reputation among locals. Interestingly though, pesticides were also described as being misused and abused to yield higher gains, creating a unique juxtaposed relationship with pesticide use. Since the khat plant requires a large amount of water to grow, the farmers can face challenges in many areas in eastern Ethiopia which have faced droughts over the years. In response, Adnan describes how khat farmers in this region use pesticides mainly during the dry season not to protect against pests, but to help the plant grow:

There is [the] legal one for the vegetables and fruits. They’re [farmers] using again the *illegal* one even to use as pesticides and that is again depending on the season when there is no rain or when the khat price is high. They don’t use the pesticide during [the] rainy season because…the main purpose of the pesticide is not to protect the insect from the khat, it is to make the plant grow up. So, I don’t know scientifically if that works. But to grow khat, the [farmers] grow khat with all the pesticides they use during the dry season.

Adnan is unsure of the mechanics as to whether or not pesticides help plants grow during dryer periods, nevertheless, this is a common belief among khat farmers who continue to use it for this purpose.

### Stigma of using pesticides

It was clear that khat users in this region preferred khat without pesticides and could often claim to tell the difference between organic khat and khat with chemicals. During the interview with the khat farmer, Ahmed, there was a white residue on the khat leaves that was initially conveyed as pesticides, however, later the farmer stated it was paint. He explains, “…[we] don’t usually use the pesticides. This, what you see [points to residue on khat] is painted to mislead thieves.” The ambivalence surrounding pesticide use made it unclear as to whether the white residue was pesticides or paint to deter potential thieves as the farmer described.

Adnan also explains how local people may be able to tell the difference between the different types of treated (using pesticides) versus untreated (organic) khat.

I may say this is good khat [points to organic khat]. If it is processed without pesticide. If you go to Jijiga [city near Harar] or the Somali Region, then you say good khat with the pesticide. So, I will teach you. It is very shiny, expensive. Some people like the expensive one because [it’s] very shiny…The other [cheaper] one maybe because of the econom[ic] status.

In this passage, Adnan ascribes economic status to which khat a person is seen purchasing or consuming. For those who can afford it, organic khat is preferred and revered as a higher-quality product. He also points out how the quality of khat is relative to where a person is located, in that in the Haramaya and Harar areas, organic khat is preferred whereas in neighboring regions and countries, the khat with pesticide may be viewed as high-quality.

### Runoff exposing people through drinking water

Due to the overuse and misuse of pesticides in this region, there is concern that pesticide runoff may be leaching into the groundwater and other water sources and also the soil. Some pesticides are found in water and soil for up to 15 years. Hasan, a microbiologist, details how he has been testing different water sources for pesticides:

So khat from the market and from the farm [have been tested]. And also the product, the sample from the underground water and the lake around this area. And we did the extraction…They [farmers] apply pesticides directly on the khat so during the rain, the pesticide is washed down and they will go down. Underground water, people directly use these waters. They make a well, they pump it out. And they use it without any treatment for drinking water.

Hasan implies how pesticide runoff in these areas can have harmful health effects by drinking from a contaminated water supply. He goes on to explain how this exposure may be one of the reasons there is a high prevalence of kidney disease in this region of Ethiopia, especially since most residents do not treat their water.

### Societal relationships around khat

Khat is widely viewed in many places in Ethiopia as a traditional and cultural element of society, however, throughout this study there were several instances where participants challenged different aspects of this idea, specifically how khat use manifests within this region. Adnan, for example, insists that there is societal pressure to chew khat:

Because it’s cultural pressure. So because we are already in the community, you already [have] the pressure from the religion, and then your peers, your friends are still doing it even though the family is not happy. But if you, if you go through with it, they tell you to reduce…Maybe some people may bother you but still, that will lead to hiding and chewing…When we are transparent and open and that is very formalized you do reduce the volume you reduce the frequency [of khat] but if you hide you take a lot because you are hiding.

Adnan postulates that there may be some cognitive dissonance, or at least contradictory beliefs surrounding societal pressures to chew khat. In that, on the one hand, there is pressure to chew khat, while simultaneously denigrating those who chew khat. He also expands upon the idea that in societies where khat is more openly acceptable, then people are less likely to abuse it than those who have to hide their khat use. Here, Adnan is perceived as justifying the normalization of khat use publicly since he goes on to mention hiding khat use also invites other risky behaviors.

### Transition of khat use

Khat use within eastern Ethiopia appears to be shifting socially to become more normalized and used for different purposes than historically or in other parts of the world. Adnan posits that “it used to be [during] the religious ceremony. Now it’s [be]coming more socially when people come together, especially in rural areas.” Adnan also describes the shifting landscape of those who chew khat in eastern Ethiopia.

But, but the rate is increasing, the number of children this day chewing and the number of children before some years chewing is completely different. Now it’s coming to the very youngest person coming to chew, especially in urban areas.

While it appeared like Adnan agreed with the normalization of khat, he did not condone khat use among young people, specifically children. Likewise, Abbas also shares his perspective on the transition of khat use but more as it has separated from religion, “It’s not to pray to God. Religious. No. Look, people may say it’s for God but no, it’s, it’s for work.” Abbas felt strongly that khat use in this region was to encourage hard work. So much so that he states, “I don’t work with the people [who] do not say that they have khat.”

### Societal pressures

Additionally, since this region is so heavily invested personally and professionally in khat, it may draw negative attention by not participating in khat chewing socially. Adnan describes how those within the community will pry and ask questions:

From a social perspective, if you are not chewing from this community - you used to chew and then you stop, they [the community] ask what happened to you. Is it from the health perspective or it is causing economic [hardship]? So, if they cannot afford it [then] you stop. ‘So why you stop?’ ‘What happened to you?’ These are the questions.

Consuming khat appears to be so inoculated at the community level that not chewing would cause unsought attention and scrutiny. Adnan implies that one must be ill or have insufficient funds to not engage in khat use, making it difficult to abstain.

### Applications of khat

The uses of khat have changed over time in this region. What was once primarily part of religious and ceremonial practices has now been transposed into more practical uses, such as studying, working, and relaxation. Abbas describes these different uses:

Yeah, yeah, yeah but most of the time in this part of Eastern Ethiopia people do not use it [for ceremony] but in central, in the northern part people who have the khat will have people who chew the khat a lot and have very high stimulant, call[ed] ‘Mirkana’. This means at nighttime you will drink some tea so it breaks [the stimulant] because it’s too late and you will break that stimulant with a cold [drink] and you will sleep. But around these areas…. chewing is for work. Chew and work, chew and work for work majorities.

*Mirkana*^1^ is commonly described as the high resulting from consuming khat; however, Abbas expresses how in this region khat is rarely used for this purpose. Because khat is the staple crop in this location and since so many livelihoods are dependent on it, many consume khat for the energy to work rather than to achieve *mirkana*. When discussing the different varieties of khat, Adnan emphasizes the energy produced is typically more important than the taste of the khat,

Actually, not tastes, but the energy, the energy inside. This stimulant. As you go for the cheaper one [khat], you need to get multi-energy. You need to take more because that’s value added. The more expensive one is more quality. More juicy. The small volume increases the quality that also [increases] the price as well again. Because the content and the energy you get from it is very…high.

Adnan underscores how energy production is a primary application of khat in this region, with the higher-priced khat providing more energy within smaller leaves - making it more potent. The use of khat for energy production appears to be a unique finding that could contribute to the physiological dependence of khat within eastern Ethiopia.

### Physiological experiences

While khat is a known stimulant, it appears to have different effects on different people. Adnan describes these differences.

[It] depends on an individual’s biology. For example, some people when they chew, they are very calm [whispers]. They don’t want to talk. They don’t want to see you talk. They need one or two silent [for] focus. While the other people become more talkative, more attractive, shy people are shy…So it depends on how people behave, some behavior they don’t like when they chew even to go to the salat [prayer]. Some people hide themselves. So it depends on an individual’s behavior in the way we learn it. Yeah. That some people will start chewing khat, they’re studying whenever they chew they need something to read.

The assumption for many is that khat primarily makes a person active, however, long-term khat use over time or in one sitting can cause lethargy or quietness, as Adnan stated. Ultimately, how khat is used is dictated at the individual level, in that it can be used to study, work a labor-intensive job, or recreationally. Abbas also reports on his accounts of chewing khat during his interview.

If I have half of this one [points to khat bundle] I can’t stand up now. This dosage is very high. It has a chemical inside, what is it called? A stimulant. So, it is more than my resistance which will make you, it will shake your body.

During the interview, due to the potency of the organic khat, Abbas was temporarily unable to stand.

### Differences in rural versus urban experiences

It was mentioned across interviews that there were differences between how khat is used within urban versus rural settings of Ethiopia. Differences appeared to stem from whether or not the area has normalized khat chewing. Adnan elucidates that “…if it is in the urban area, you see maybe the children on the street are homeless children. They buying. They’re buying, they’re chewing.” He goes on to state, “Away in a rural area nobody is going to buy because everybody has their own field.” This implies that accessibility to khat may impact whether it has become more normalized within a community.

### Access to healthcare

Access to healthcare was described during an interview with a local government worker, Mariam, who collects surveillance data at the household level. She explained that access to healthcare within the region is very limited and the number of trained medical providers are only at the hospital level in surrounding areas. She states:

The health post was the level there’s no physician, but at the hospital level [they do] … and then because of this level of health post-secondary challenges so that our delivery physician people - who have a background degree level, provide service at the closest hospital.

Given the limited health services, as well as other resources, in this area, Mariam cautions against risky behaviors that could lead to complications within a medical setting. During this interview, she discussed pregnant women giving birth without sufficient medication in somewhat unsterile environments. Given the pervasive nature of khat use within these settings, there appears to be a high risk of medical complications that could arise.

## Discussion

Khat, a natural stimulant cultivated and commonly used in the Haramaya District and across eastern Ethiopia, is recognized as an addictive substance primarily consumed for its stimulating properties to aid in performing daily tasks [3, 4]. As such, research has noted the pervasive presence of khat within communal socialization [7], economic stability [3, 17], and physiological maintenance [13]. Though previous research has identified the significance of khat within this region’s societal functions, this study is the first to capture and extract the connotation and interpretive meaning of khat among the region’s residents by centering around their lived experiences. Thus, this study aimed to gain a deeper understanding of the livelihoods, experiences, and relationships of Haramaya District residents with khat through a qualitative interpretative phenomenological analysis.

Historically, khat consumption was restricted to Islamic royal and religious figures and limited to Ethiopia’s Harar region. However, by the beginning of the 20th century, chewing and cultivating khat spread across various communities, including Muslims and Christians [28]. While khat continued to be associated with religious or spiritual significance in many Ethiopian regions, its cultivation saw a significant expansion in the Hararghe region by the 1950s, with the area of land devoted to its growth increasing from 2,996 hectares to 7,009 hectares [29]. By the 2020s, khat had transformed from merely a substance consumed for its euphoric effects, known as *mirkana*, to a vital economic commodity many families depend on for their income. It does not go without stating that there still exists a tension between khat being used for spiritual use versus economic - as indicated by Adnan and Abbas. Additionally, there is the distinction between khat being used spiritually versus religiously. While khat may have originated among Muslims, it has been propagated to all social groups regardless of religion, age, or gender [28]. Because khat is widely considered spiritual, rather than faith-based, intervention development may need to focus on abstract or cognitive processes related to higher-being beliefs rather than religious practices.

Our findings illuminated a prevalent stigma associated with khat use, manifesting in ways distinct from those previously documented. More specifically, stigma was associated with the underlying addiction that perpetuates its use rather than khat itself. Though researchers have established khat as a pharmacologically addictive stimulant [30], residents expressed a taboo nature behind the word “addiction” to the point where “dependence” was the preferred term in most conversations. The stigma associated with substance use is not a novel concept and is often cited as denial within addiction [31, 32]. Interestingly, denial often exists in an intrapersonal landscape of cognition, not a communal approach toward use. Within our findings, the noted societal normalization and inculcation of khat use creates a large-scale collective denial among residents [33]. Though the denial is shared across communities, it is seemingly out of individual self-preservation to maintain a degree of economic stability and societal integrity. The unique interplay between communal practice and individual preservation creates a cyclical effect of using khat to supplement energy to farm khat and then sell or stimulate further work on their farm.

**Fig. 1:**
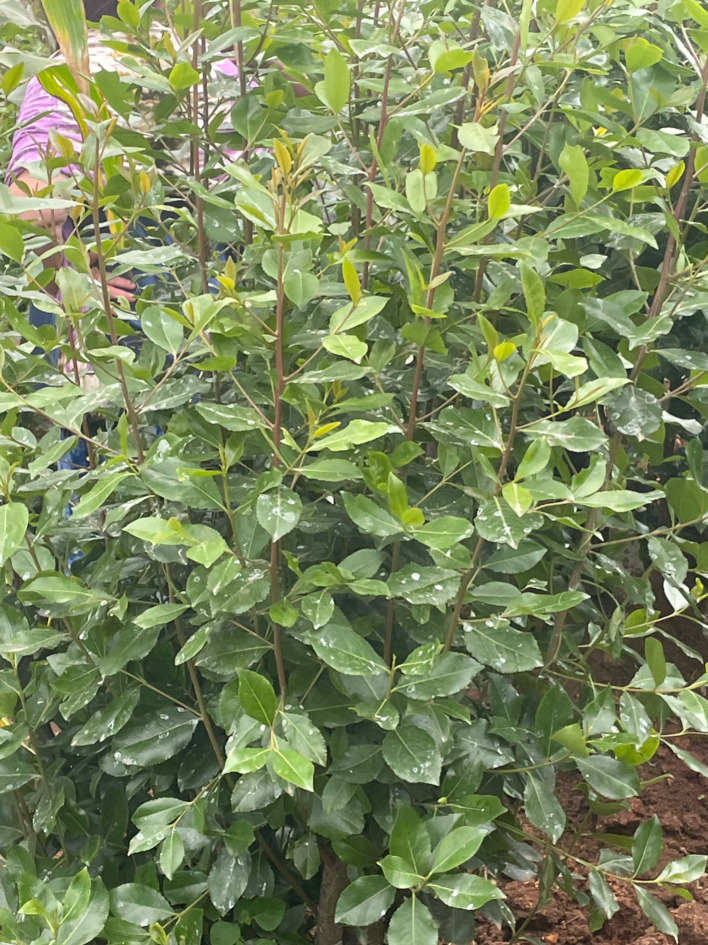
Khat with suspected pesticides

Consequently, this cyclical nature within the Haramaya District has modified the relationship with khat to deviate from ‘traditional’ to transactional and serve as a mechanism for earning. This metamorphosed relationship has created another thread of stigma associated with agricultural practices, emphasized in the salient theme, Pesticide Use. This theme depicted a contentious topic for several participants due to khat farmers not wanting their farms linked with using pesticides due to the communal perception that chemically-treated khat is considered lesser quality than organic khat as well as the source of various health-related issues. However, pesticides were also described as being misused and abused in order to yield higher gains, creating a unique juxtaposed relationship with pesticide use. Similar dichotomies exist within other regions with ongoing discussions regarding sustainability practices versus environmental dangers, health consequences, and ethical issues associated with pesticide use [34]. Boiral and colleagues [34] found that to address the stigmatization of pesticide use, common practices included denial, claims of victimhood, defense of social justice, and rural traditions. Denial parallels one of our participant’s anecdotes of conveying pesticides as paint (Fig. 1). He further explains that organic khat is preferred and revered as a higher-quality product, seemingly inconsistent with some literature. In Finnish agriculture, Lähdesmäki and colleagues [35] investigated the early stigmatization resulting from deviating from conventional practices to organic farming. This is particularly relevant to our findings as one participant pointed out how the quality of khat is relative to where a person is located, noting that in neighboring regions and countries, khat with pesticides may be viewed as high quality. This inconsistency of what is deemed stigmatized inherently continues to entangle the relationships and connotations associated with khat within this community.

Moreover, the involvement of women in selling khat in Haramaya and Harar defies traditional gender norms throughout the rest of Ethiopia, as women actively participate in an industry traditionally dominated by men. This unique gender dynamic, however, has unintended consequences, contributing to increased malnutrition within households [36]. Studies have shown that women’s growing and selling of khat leads to elevated responsibilities, diverting their attention from traditional gendered roles, such as child-rearing [37]. According to Mechlowitz [37], the cumulative workload was the indicator that contributed the most to women’s disempowerment. Despite women’s roles and workloads not being measured within the current study, the intersection of women’s participation in the khat trade and the resulting nutritional challenges underscores the need for a nuanced understanding of how gender norms and economic activities influence household well-being in Haramaya.

The current study has several limitations. With only six participants, its focus on khat use in Haramaya, Ethiopia, does not provide a comprehensive view of all individuals who have experience with khat consumption or cultivation in the region. Future studies would gain from expanding the sample size and including more female participants as well as those from various socioeconomic backgrounds. Moreover, this study is qualitative and idiographic, designed not to generalize but to capture the intricate experiences of a limited group of participants.

## Conclusion

These findings are the first insight into how the people within eastern Ethiopia make sense of their personal experiences relating to khat. The participants’ responses provided insight into the transactional nature of khat that has now been embedded within society, with the substance influencing traditional gender roles to promote the sale of khat while the traditional applications of khat in religious and spiritual settings are waning. As such, future population-based interventions occurring in the Haramaya District of eastern Ethiopia must consider these individuals’ lived experiences and recognize khat’s power over the community’s livelihood.

## Data Availability

The data that support the findings of this study are openly available at: https://data.mendeley.com/datasets/ts92kvy69d/1.

## Footnotes

1 Perspectives of how other regions break mirkana was not validated in this study
